# Congenital Cytomegalovirus Reference Material: A Content Analysis of Coverage and Accuracy

**DOI:** 10.1007/s10995-013-1275-0

**Published:** 2013-04-26

**Authors:** Rosemary Thackeray, Allison Wright, Katherine Chipman

**Affiliations:** Department of Health Science, Brigham Young University, 221 Richards Building, Provo, UT 84602 USA

**Keywords:** Cytomegalovirus, Prevention, Websites, Pregnancy, Books

## Abstract

Congenital cytomegalovirus (CMV) is the leading cause of birth defects and developmental delays in the United States. However, only 13–22 % of women in the United States have heard of CMV. This research assessed (1) the quantity and accuracy of CMV information included on pregnancy-related websites and reference books, and (2) whether CMV information was included less often than information about other birth defects or infections. A content analysis of 37 pregnancy reference books and seven websites was conducted. The data collection instrument represented categories describing CMV, transmission, and prevention. CMV subject matter experts at the Centers for Disease Control and Prevention reviewed the instrument. Each book and website was coded independently by two different coders. Twenty-one reference books and seven websites included CMV content. CMV was less likely to be included as a topic than other infections or birth defects. There were fewer sentences about CMV than toxoplasmosis, Down syndrome, or HIV. Book length was associated with increased likelihood of including CMV. How to prevent CMV transmission was discussed only half the time. Though limited, nearly all the CMV information was accurate. Pregnancy-related reference books and websites contain limited CMV information. Books are less likely to include CMV as compared to other infections and birth defects. Most of the CMV information is accurate. There is inadequate coverage given to prevention of CMV transmission, which may contribute to CMV remaining a continued leading cause of birth defects in the United States.

## Introduction

Congenital cytomegalovirus (CMV) is the leading cause of birth defects and developmental delays in the United States [1]. More children are affected by CMV disease than other well-known congenital conditions such as Down syndrome or fetal alcohol syndrome [2]. The estimated prevalence of congenital CMV infection differs by research study, perhaps due to the varying definition of what is considered a symptomatic infection [1]. In a review of 27 different study groups throughout the world, the combined birth prevalence was estimated at 0.64 % [1]. In this same study, of those infants born with CMV, only 0.07 % were symptomatic. Another review of 15 CMV studies conducted across the globe revealed a birth prevalence of 0.7 % and a symptomatic rate of 12.7 % [3]. Currently, the Centers for Disease Control and Prevention estimate that in the United States, approximately 30,000 infants are born each year with CMV infection [4]. In addition, approximately 5,000 of these infants will develop permanent disabilities [4]. Permanent sequelae include sensorineural hearing loss [5], death, vision loss, developmental disabilities and cognitive impairments [1, 3].

Cytomegalovirus is transmitted from child-to-mother, primarily through bodily fluids of urine and saliva [6]. Recent research has shown that the virus can also remain on certain absorbent surfaces such as cotton cloth, crackers and plywood, thus providing additional transmission routes [7]. Once infected, a child can shed the virus for several years [8]. Thus, women associating with or having close contact with young children, either domestically or in the workplace, are particularly susceptible to contracting the virus and passing it along to their unborn infant [9]. Routine testing for CMV is not recommended and there is not a vaccine available [10].

Prevention of CMV transmission between the mother and child is focused on improving hygienic practices. This includes routine hand washing, not sharing food or utensils, and not kissing the child on the lips [2]. Prevention-based interventions have focused on education and counseling, particularly in health care settings [11, 12].

Awareness of CMV is relatively low among women. Approximately 13–22 % of women in the United States have heard of CMV [13–16]. A study in France showed that 74 % of women who received services at a particular hospital were aware of CMV [17]. In Singapore, 20 % of women have heard of CMV [16]. One reason for this general lack of awareness may be due to a dearth of information about CMV that women of child-bearing age receive. Healthcare providers are a natural source of pregnancy-related information. Sixty-nine percent of women in the United States, ages 18–44 reported visiting an ob/gyn within the last year [18]. Despite the recommendation that CMV is part of the health promotion counseling women receive [19], less than half of obstetricians/gynecologists in the United States report counseling their patients about how to prevent CMV infection [20].

In addition to a healthcare provider, reproductive-age women in North America seek information from other sources. These include the internet [21–23], discussion forums [24], family members, and books [23, 25]. According to Read et al. [26], pregnancy books and the popular press contain very little, if any, information about CMV and its prevention, though the statement was based on the authors’ observations and not actual data.

## Objectives

The purposes of this research were to (1) perform a content analysis to assess the quantity and accuracy of CMV information included on pregnancy-related websites and reference books, and (2) assess whether CMV information was included less often than information about other birth defects or infections.

## Methods

The authors defined leading pregnancy reference books as the top 100 books based on sales and/or average customer reviews of pregnancy and childbirth books on three websites: Amazon.com, BarnesandNoble.com, and Walmart.com. In addition, searches on Google Books and Google Shopping were completed using the terms pregnancy and pregnancy books. Initial inclusion criteria were that the book was published between January 2001 and December 2012 and it was written in the English language. This resulted in a list of 800 books. Each book description was reviewed to verify that it met the initial inclusion criteria. Duplicate book references and earlier editions were eliminated. Next, novels, specialty books (e.g., multiple births), journals or calendars, books about rearing children, and books authored by an organization or individuals without credentials were excluded. The final sample was 37 books. A hard-copy or e-book copy of each book was obtained. For each book, electronic text searches for the words CMV or cytomegalovirus were conducted; the terms were found in 21 books (57 %) [27–45].

To identify pregnancy-related websites, searches for pregnancy were conducted on search engines Google, Yahoo, MSN, and Dogpile. The seven websites that appeared on all four search lists were selected, including: pregnancy.org, medicinenet.com, babycenter.com, webmd.com, whattoexpect.com, mayoclinic.com, and everydayhealth.com. Each website was searched using the terms CMV and cytomegalovirus and reviewing the A–Z topic lists. The inclusion criteria were that the website had to mention congenital CMV, or pregnancy and CMV.

Content analysis methodology was used based on previous research related to assessing accuracy and quality of health-related content on the internet [46–48]. The research team developed a data collection instrument representing categories related to describing CMV, how CMV is transmitted, and how to prevent infection. The instrument was reviewed by CMV subject matter experts at the Centers for Disease Control and Prevention (CDC). Each variable was coded as yes or no. Each book and website was coded independently by two different coders; discrepancies were discussed and resolved by consensus. Agreement ranged from 94 to 95.4 %.

To assess accuracy of CMV information contained in books, actual text concerning seven variables about CMV statistical rates and prevalence were recorded: (1) the percent of women who have antibodies to CMV before getting pregnant, (2) the percent of women who are seronegative who will get a primary infection during pregnancy, (3) percent of primary maternal infection that will lead to a fetal infection, (4) the number of children born each year with CMV infection, (5) number of child deaths from CMV infection, (6) the number of children who experience permanent disabilities from CMV infection, and (7) the likelihood of passing CMV to your infant if a reinfection occurs during pregnancy. Accuracy results were confirmed with CMV subject matter experts at the CDC.

To compare the quantity of CMV information to other congenital infections or birth defects, seven topics were identified for evaluation: toxoplasmosis, listeriosis, group B strep, HIV, Down syndrome, fetal alcohol syndrome, and spina bifida. These were chosen based on the preventable nature of the infection or birth defect (with the exception of Down syndrome), the potential for fetal complications, and the publics’ general level of awareness of these topics [13].

Each of the original 37 pregnancy reference books was assessed to determine if they included content about the additional infections and birth defects. Next, each of the 21 books that contained CMV information was coded for five areas across all topics: permanent outcomes, prevention (except for Down syndrome), and rates of infection or prevalence, disability, and death. We chose to compare only within books that had CMV content to see if the amount of content, not the presence of content, was significantly different than other infections or conditions. The coders were in agreement 85.4 % of the time; discrepancies were resolved by consensus.

## Results

Just over half (n = 21; 57 %) of the 37 reference books and each of the seven websites included CMV content. For books, there was a statistically significant difference between the congenital infections and birth defect topics and the likelihood of the topic being included (χ^2^ = 31.608, *p* = .00). Examination of standardized residuals revealed that CMV was less likely to be included (Cramer’s V = .33). The remainder of the results refers to the books and websites that included CMV.

There was a statistically significant difference in the mean number of book pages and whether it included CMV with longer books being more likely to include CMV information (t = 3.37; *p* = .002). No books had a specific chapter devoted to CMV. CMV was most commonly found in a section about infections and was more often located in the index than the table of contents (Table 1). Six of the seven websites had a specific page dedicated to CMV.

**Table 1:** Where the topic was found in pregnancy reference books

Topic	Table of contents % (N)	Index^a^ % (N)	Text search % (N)
CMV	19.0 (4)	90 (18)	100 (21)
Down syndrome	9.5 (2)	90 (18)	95.2 (20)
Fetal alcohol syndrome	0 (0)	75 (15)	90.5 (19)
Group B strep	19.0 (4)	85 (17)	90.5 (19)
HIV	14.3 (3)	95 (19)	100 (21)
Listeria	19.0 (4)	80 (16)	100 (21)
Spina bifida	0 (0)	75 (15)	90.5 (19)
Toxoplasmosis	19.0 (4)	95 (19)	100 (21)

^a^Only 20 books had an index

The amount of CMV content in each book and on each website varied. Books contained a mean of 10.8 sentences about CMV (range = 0–28; the book with no sentences had one chart). Charts with CMV information were included in less than one quarter (23.8 %) of books; no more than one chart was in each book. Websites included a mean number of 40.7 sentences (range = 4–126). None of the websites contained CMV-related charts. No books recommended where to go for more information. One website recommended going to the CDC’s CMV section for more information and provided a link.

Less than half of books (n = 10) and just over half of websites (n = 4) discussed any risk factors for maternal CMV infection. Seventy percent of these books (n = 7) and 50 % of these websites (n = 2) mentioned, in general, that contact with young children is a risk factor for CMV. They identified specific risk factors of being a mother of a toddler (books: n = 3; 30 %; websites: n = 1; 25 %), having an occupation involving daily contact with children (books: n = 8; 80 %; websites: n = 3; 75 %), and working in a healthcare occupation involving contact with CMV patients (books: n = 5; 50 %; websites: n = 3; 75 %).

Over half of the books (n = 13) and all of the websites mentioned modes of transmission. Eleven of these books (84.6 %) stated that CMV could be passed from mother to the fetus, while only six of the seven websites (85.7 %) stated this fact. However, two-thirds of books (n = 7) discussed how CMV can be transmitted through contact with secretions of an infected person while 71.4 % (n = 5) of websites did so. Only 9.5 % of the books (n = 2) but 71.4 % (n = 5) of websites stated that maternal CMV infection leads to lifelong latent infection. Similarly, only 28.6 % of the books (n = 6) but 71.4 % of websites (n = 5) stated that maternal recurrent infections or reinfection can occur.

Overall, prevention behaviors were included in only 12 books (57.1 %) and four websites (57.1 %). Within these books and websites, the inclusion of specific prevention recommendations varied and tended to focus primarily on washing hands with soap and water (Table 2).

**Table 2:** Behaviors recommended to prevent CMV transmission

Recommended prevention behaviors	Books N (%)	Websites N (%)
Good personal hygiene	5 (41.6)	2 (50)
Clean toys, countertops, and other surfaces that come in contact with urine or saliva	0 (0)	0 (0)
Do not kiss on or near the mouth	0 (0)	2 (50)
Do not share cups, plates, utensils, toothbrushes, or food	2 (16.6)	3 (75)
Do not share towels or washcloths	0 (0)	0 (0)
Practice safer sex if not in a mutually monogamous relationship; use condoms and avoid oral sex	2 (16.6)	2 (50)
Thoroughly wash hands with soap and water	11 (91.6)	4 (100)
Thoroughly wash hands with soap and water after contact with bodily fluids (urine, saliva, feces)	4 (33.3)	2 (50)
Thoroughly wash hands with soap and water after diaper changes or helping kids use the restroom	5 (41.6)	4 (57.1)
Thoroughly wash hands with soap and water after feeding or bathing child	1 (8.3)	0 (0)
Thoroughly wash hands with soap and water after handling child’s toys	0 (0)	0 (0)
Thoroughly wash hands with soap and water after wiping child’s runny nose or drool	2 (16.6)	2 (50)

Of those books (n = 12) and websites (n = 4) that recommended prevention

Few books (n = 3; 14.3 %) included recommendations for when a woman should see a doctor if she is concerned about CMV while 71.4 % (n = 5) of websites did so. Both books and websites indicated that the woman should see a doctor if she is exposed to CMV or thinks she is at risk, or if she notices symptoms of CMV such as a fever or other flu-or mono-like symptoms. All of the websites and 61.9 % (n = 13) of books mentioned symptoms for pregnant women; just over half the time (n = 11; 52.4 %) books indicated that women are usually asymptomatic while all the websites indicated the same. Both books and websites described symptoms as flu-like (books: n = 5; 38.4 %; websites: n = 2; 28.6 %) or mono-like (books: n = 3; 23.0 %; websites: n = 5; 71.4 %).

Both books and websites included guidance for screening among women at the same rate (42.9 %). The main point of these recommendations was that CMV testing is not routine for pregnant women but that they should ask their doctor about screening if they suspect they have been exposed to CMV or if they are at a higher risk of CMV infection due to their profession or having frequent contact with young children.

One third of books (n = 7; 33.3 %) and just over half of websites (n = 4; 57.1 %) discussed how CMV is diagnosed in fetuses, specifically amniocentesis and ultrasound exams. Eight books (38 %) and two websites (28.6 %) referenced maternal or infant treatment options for CMV. Six books (28.6 %) and one website (14.3 %) stated that no treatment is currently available and one book stated that there is no cure. Prenatal CMV hyperimmunoglobulins (HIG) and ganciclovir were each mentioned in one book, antiviral medication was mentioned in three books and one website, but valacyclovir was not mentioned in any of the books or websites. Only two books (9.5 %) and two websites (28.6 %) mentioned that there is not currently a vaccine.

Five books (23.8 %) and four websites (57.1 %) stated that most infants are asymptomatic at birth. Nineteen percent of books (n = 4) and 42.9 % of websites (n = 3) discussed the potential that infants may or may not exhibit symptoms at birth and likewise may or may not develop complications later. The mention of permanent outcomes was common among books and websites (Table 3). The most frequent idea expressed in these statements was that CMV is the most common congenital infection in the United States and the leading cause of congenital deafness.

**Table 3:** Mention of CMV rates and permanent outcomes or complications

	Book N (%)	Web N (%)
Statistics		
Percent of women who have antibodies to CMV before pregnancy	4 (19.0)	2 (28.6)
Percent of primary infections among pregnant women	5 (23.8)	2 (28.6)
Percent of primary infections that lead to fetal infections	10 (47.6)	3 (42.9)
Number of children born with CMV each year	5 (23.8)	1 (14.3)
Number of deaths each year from congenital CMV infection	1 (4.8)	0 (0)
Number of permanent disabilities each year from congenital CMV infection	8 (38.1)	2 (28.6)
Chance of passing CMV to the fetus during pregnancy if a CMV infection recurs	4 (19.0)	3 (42.9)
How common CMV is among the general population	9 (42.9)	3 (42.9)
Likelihood of maternal CMV reactivation or reinfection	0 (0)	0 (0)
Rate of congenital CMV infection and complications compared with other congenital diseases	9 (42.9)	4 (57.1)
Permanent outcomes		
Central nervous system damage	3 (14.3)	3 (42.9)
Hearing loss	17 (81.0)	6 (85.7)
Mental retardation	11 (52.4)	5 (71.4)
Microcephaly	3 (14.3)	2 (28.6)
Miscarriage/stillbirth/death	9 (42.8)	3 (42.9)
Motor disabilities	2 (9.5)	2 (28.6)
Seizures	0 (0)	1(14.3)
Vision impairment	12 (57.1)	5 (71.4)

In all but three areas, CMV information was accurate. First, nine out of ten statements about the percent of primary maternal infections that will lead to a fetal infection were correct. Second, four of the eight statements related to the number of children who experience permanent disabilities each year from congenital CMV infection were inaccurate. Third, there were seven books that made statements indicating that a woman could develop or possess CMV immunity, a concept that is associated with reinfections and is not accurate. All website statements were accurate. However, while accurate, the frequency of these facts was low (Table 3).

In comparing CMV to other congenital infections and birth defects, each of the 21 books that included CMV also included information about the other topics (Table 4). There was a significant difference in the mean number of sentences between the eight topics (F = 2.84, *p* = .008). The books contained a mean of 10.8 sentences about CMV which was half as many sentences, on average, as the other topics (range = 15.6–30.4). Specifically, the mean number of sentences for CMV was significantly less than toxoplasmosis (t = 3.32, *p* = .00, $\bar{x}$ = 28.8), Down syndrome (t = 2.82, *p* = .01, $\bar{x}$ = 30.4), and HIV (t = 2.54, *p* = .02, $\bar{x}$ = 20.6). Books were less likely to include information about prevention of CMV and spina bifida than about fetal alcohol syndrome, group B strep, HIV, listeria, or toxoplasmosis (χ^2^ = 25.5, *p* = .00, Cramer’s V = .416).

**Table 4:** Comparison of CMV with other infections and birth defects in pregnancy reference books

Topic	Average number of sentences N (SD)	Rates N (%)	Prevention N (%)	Permanent outcomes N (%)	Charts ± N (%)
CMV	10.81 (8.50)	18 (85.7)	12 (57.1)	17 (81.0)	5 (23.8)
Down syndrome	30.43 (30.68)	16 (76.2)	N/A	14 (66.7)	9 (42.9)
Fetal alcohol syndrome	15.76 (12.53)	7 (33.3)	19 (90.5)	18 (85.7)	4 (19.1)
Group B strep	19.43 (20.01)	17 (81.0)	18 (85.7)	17 (81.0)	3 (14.3)
HIV	20.62 (15.52)	15 (71.4)	20 (95.2)	20 (95.2)	5 (23.8)
Listeria	16.66 (12.90)	13 (61.9)	21 (100.0)	16 (76.2)	4 (19.1)
Spina bifida	15.81 (12.51)	10 (47.6)	16 (76.2)	12 (57.1)	6 (28.6)
Toxoplasmosis	28.76 (23.32)	10 (47.6)	21 (100.0)	20 (95.2)	6 (28.6)

±any type of graph or grid that mentioned CMV

Rates—any mention of incidence, prevalence, infection rate, transmission rate, rate of permanent disabilities

Prevention activities—any mention of actions that will help to prevent the spread of the infection or the development of the birth defect

Permanent outcomes—lasting complications or effects as a result of the infection or birth defect

Number of sentences—complete sentences (determined by punctuation) about or related to topic that were not in a table or chart form

Charts—data, numbers, or text were presented in a graphical form, in a table, or in separate text box

## Discussion

This is the first study to assess the quantity and accuracy of CMV information contained in pregnancy-related reference books and on websites. It also compared the quantity of CMV information to other birth defects and pregnancy-related infections. CMV was not included in all pregnancy books, but on all the websites. Books were less likely to include CMV than other birth defects or congenital infections. Though limited, the CMV-related information in both books and websites was nearly always accurate. These data confirm the assertion of Read et al. [26] that pregnancy books contain little information about CMV and its prevention.

The amount of CMV information in books and on websites was limited. For books, the amount of CMV coverage was statistically different than for the pregnancy topics of toxoplasmosis, Down syndrome, and HIV. Low average sentence count indicates that comprehensive information about what CMV is, how it is transmitted, how to prevent transmission, and the potential outcomes are lacking. The number of sentences devoted to CMV is alarming considering that CMV causes more birth defects than other more well-known conditions.

It was less common for pregnancy-related books to contain CMV information than it was for websites to do so. Just over half of books included CMV. In these books, CMV was mostly likely to be found in the index and not in the table of contents or a specific section dedicated to it. This is probably due to how books are structured, which is often by trimester and not specific topics. Because CMV information is limited and is not in a prominent location, a woman would have to be specifically searching for CMV, otherwise she may overlook it.

Discussion about risk factors for and transmission of CMV infection were not universal. In particular, identifying close contact with young should be paramount, as this is how the CMV virus is transmitted. Additionally, while reference materials described how CMV is transmitted, they were missing the key point of secretions from bodily fluids of an infected person. The lack of education and awareness about these two facts alone place women at increased risk for contracting CMV and passing it to their infant.

Also of concern is the low number of books and websites that addressed prevention of CMV. Prevention strategies were included less often for CMV than for the other birth defects and infections, except for spina bifida. It is relatively easy to avoid CMV transmission and prevention is critical since there is not a vaccine or other cure. However, general mention of how to prevent CMV transmission is discussed only about half of the books and websites. One of the most effective behaviors for preventing CMV transmission, as well as other infections, is hand washing [49]. Other recommended preventive behaviors such as not sharing cups or eating utensils, not kissing young children on the lips, or not sharing towels or washcloths are rarely mentioned. These additional hygienic related behaviors are less likely to be included perhaps due to concerns about the effectiveness of the interventions that are aimed at encouraging women to follow the recommended course of action [2].

With few exceptions, the information about CMV, as assessed by the seven selected variables, was accurate. However, the frequency with which these variables were mentioned was low, therefore giving the reader an inaccurate picture of the true risk and potential outcomes of CMV infection. One area of inaccuracy that raised concern was related to statements that a woman can be immune to CMV once she has been infected. While a woman can develop CMV antibodies, it does not prevent her from being reinfected with a new strain of CMV [50]. This may give women a false sense of security concerning her risk and the risk to her unborn child.

Although pregnancy related books and websites contain limited CMV information, if any at all, low CMV awareness among women may only be partially attributable to this lack of information. There was no difference in the amount of CMV information in books compared with fetal alcohol syndrome, group B strep, listeria, or spina bifida [14]. However, women’s awareness of these other topics is higher than CMV. The discrepancy may also be due to women being more aware of other conditions as a result of routine screening that is offered to pregnant women for conditions such as Down Syndrome [51]or Group B Strep [52], or as a result of public awareness campaigns such as for folic acid for preventing neural tube defects, including spina bifida [53].

## Limitations

While the number of sentences about CMV was selected as an indicator of quantity, it may be an over-representation of the true value. Health literacy guidance suggests that writing text for lower literacy level will include fewer multiple syllable words and shorter sentences [54]. Reference material written this way, as was the case in some of the pregnancy books, may artificially inflate the quantity of information represented as there are multiple short sentences.

Assessing accuracy of information was hampered by the use of phrases such as “few,” “very low risk,” and “small” to describe rates, percentages, and potential risk. These words, in contrast to that of actual numerical values, may make it difficult for women to correctly asses their risk or the risk to their unborn child. These descriptive words may convey that CMV is not very important topic for which pregnant women should be concerned.

There are limitations to how books and websites were selected for inclusion. Books were selected based on sales rank and customer rating. However, this does not mean that they represent all pregnancy reference books. Similarly, there was no way to know which pregnancy websites are the most popular among women. The authors looked at sites that were at the top of four different search engine lists. However, it is possible that the seven sites selected may not be representative of the most viewed websites for pregnancy or congenital CMV.

## Conclusions

Pregnancy-related reference books and websites contain limited CMV information. While not comprehensive, most of the CMV information is accurate. There is inadequate coverage given to prevention of CMV transmission, which may contribute to CMV remaining a continued leading cause of birth defects in the United States. To address this paucity of information and the lack of awareness will require a multifaceted approach among maternal and child health care professionals and advocates. As mentioned previously, healthcare providers play a central role in provision of care, and thus should routinely inform women about the risk of CMV and how to prevent transmission. Public awareness campaigns similar to those conducted by the March of Dimes to increase folic acid intake to prevent birth defects [55], can be effective. In addition, developing partnerships between advocacy organizations such as stopcmv.org, the Centers for Disease Control and Prevention, and the authors of websites and books to promote awareness and ensure accuracy of information is necessary.
